# Characteristic of Paraspinal Muscle Change in Coronal Sub‐type of Degenerative Lumbar Scoliosis and its Potential Clinical Significance

**DOI:** 10.1111/os.14185

**Published:** 2024-08-08

**Authors:** Zhenguo Shang, Hengrui Chang, Jiaxin Xu, Wenyuan Ding, Hui Wang, Di Zhang

**Affiliations:** ^1^ Department of Spinal Surgery Hebei Medical University Third Hospital Shijiazhuang China; ^2^ Hebei Joint International Research Center for Spinal Diseases Shijiazhuang China

**Keywords:** Asymmetric degree, Bony structural parameters, Degenerative Lumbar scoliosis, Fat infiltration, Paraspinal muscle

## Abstract

**Objective:**

Clarifying paraspinal muscle (PM) change in degenerative lumbar scoliosis (DLS) is positive to evaluate the progression of scoliosis. This research compares the characteristic of PM change among different coronal sub‐types of DLS and explores its potential clinical significance.

**Methods:**

A total of 84 DLS patients between June 2019 to December 2021 were retrospectively analyzed. Patients were classified into three types based on the coronal balance distance (CBD): Type A, CBD <3 cm; Type B: C7 Plumb Line (C7PL) shifted to the concave side of the curve, and CBD >3 cm; Type C: C7PL shifted to the convex side of the curve, and CBD >3 cm. Fat infiltration rates in the multifidus (MS) and erector spinae (ES) at the apex of the main and fractional curves, and spinopelvic parameters were analyzed statistically. Pearson's or Spearman's correlation was applied to analyze the correlation between asymmetric degree of PM change and these parameters in three types.

**Results:**

There were 62 cases with coronal sub‐Type A, 6 cases with Type B, and 16 cases with Type C. Patients in Type B and C demonstrated higher fat infiltration in MS on the concave side of both the main and fractional curves when compared to those in Type A. The asymmetric degree of ES change was positively correlated with CBD at the apex of the main curve in Type B and at the apex of the fractional curve in Type C respectively, and that of MS was positively correlated with apical vertebral rotation, while negatively strong‐correlated with pelvic incidence and sacral slope in Type C.

**Conclusion:**

PM fatty infiltration presented difference among varied coronal sub‐types of DLS patients. The CBD in Type B and C patients was correlated with the asymmetric degree of ES change.

## Graphical abstract

In conclusion, the degree of fatty infiltration in paraspinal muscles differs among various coronal subtypes of degenerative lumbar scoliosis (DLS) patients. The coronal balance distance (CBD) in patients with coronal imbalance is correlated with the degree of asymmetry in erector spinae changes. The strengths of this study are as follows. First, this study reports the degree of fatty infiltration in paraspinal muscles differs among various coronal subtypes of DLS patients for the first time. Second, it clarifies that the CBD in patients with coronal imbalance is correlated with the degree of asymmetry in erector spinae changes. In addition, we also discuss the potential relationship between anatomical location and function of PM and spinal deformity.

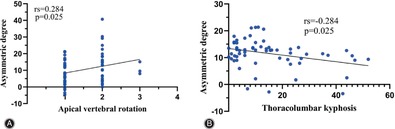

## Introduction

Degenerative lumbar scoliosis (DLS) is a spinal condition where the spine curves more than 10° in mature individuals, following the Cobb method.^1^ DLS is a complex three‐dimensional spine deformity, primarily affecting the lumbar region.^2^ Individuals with DLS often experience low back pain and leg pain due to issues like spinal stenosis and spondylolisthesis, significantly impacting their physical and mental well‐being.^3^ DLS is a noteworthy condition, with an 8.85% incidence in western countries and 13.3% in the Chinese Han population, making it a concern in our aging society.^4^, ^5^

Previous research has shown that the asymmetrical degeneration of paraspinal muscles can lead to the development of DLS.^6^ However, it remains unclear whether spinal deformities influence the pathology of paraspinal muscles (PM) or if PM issues are a result of DLS. Paraspinal muscles play a vital role in stabilizing and controlling the lumbar spine.^7^ Studies have revealed increased fatty infiltration in both the erector spinae and multifidus muscles, with the latter being particularly affected in severely degenerated lumbar regions, especially among women.^8^, ^9^, ^10^

Several studies have indicated associations between lumbar lordosis, pelvic incidence, pelvic tilt, and sacral slope with fat infiltration or atrophy of paraspinal muscles.^11^, ^12^ Jiang *et al*. and Xie *et al*.^13^, ^14^ demonstrated asymmetric morphological changes (fat infiltration) in paraspinal muscles between the concave and convex sides in patients with spinal scoliosis. Histological examinations have consistently shown a correlation between muscle fat detected by magnetic resonance imaging (MRI) and intraoperative specimens of paraspinal muscles.^15^

Recently, asymmetric atrophy and fat infiltration in the multifidus were found to be associated with a significant decline in quality of life as they increased.^16^ The ability of a patient to maintain local alignment was more influenced by muscle condition than the correction of sagittal parameters.^17^ Higher fatty degeneration and lower muscularity have been reported as risk factors for proximal junctional kyphosis and failed intra‐operative neurophysiological monitoring waveforms, independent of distal junctional problems.^18^, ^19^, ^20^ The paravertebral muscle and psoas were responsible for the maintenance of global spinal alignment in patient with DLS.^21^ While paraspinal muscles in the lower lumbar segments were likely to be primarily involved in maintaining local alignment.^17^ The progression of lumbar scoliosis could be further exacerbated by an imbalance in bilateral lumbar force caused by different degrees PM degeneration.^22^ Therefore, it seems important to capture the characteristic of PM change in coronal sub‐type of DLS and its potential clinical significance considering the entire lumbar spine to treat patients with DLS.

However, there is a shortage of published studies that have quantitatively evaluated paraspinal muscles in the coronal subtype of DLS, and the relationship between paraspinal muscles and deformity parameters in DLS remains uncertain. This study aims to: (i) elucidate the characteristic of paraspinal muscle change in coronal sub‐type of degenerative lumbar scoliosis; and (ii) explore its potential clinical significance.

## Materials and Methods

### Study Design and Population

This retrospective study was performed in line with the principles of the Declaration of Helsinki. Approval was granted by the Ethics Committee of Hebei Medical University Third Hospital. (protocol code 2022‐094‐1; 2022/10/25). This study was conducted at Hebei Medical University Third Hospital from June 2019 to December 2021. Inclusion criteria consisted of: (i) diagnosis of DLS with a minimum follow‐up of at least 1 year; (ii) age ≥ 45 years; (iii) having undergone lumbar MRI, lumbar X‐ray, and global spinal X‐ray examinations; and (iv) having a primary curve and a fractional curve. Exclusion criteria included: (i) a history of previous lumbar surgery; (ii) prior diagnosis of adolescent idiopathic scoliosis (AIS); (iii) presence of bone tumors or systemic chronic wasting diseases that could lead to paravertebral muscle atrophy; and (iv) missing imaging data.

106 DLS patients were initially reviewed, 13 patients were excluded and 9 patients had missing data; 84 patients were analyzed. Patients were categorized into three types (Type A, Type B, and Type C) based on the coronal balance distance (CBD).^23^ CBD was defined as the horizontal measurement between the C7 plumb line (C7PL) and the central sacral vertical line on coronal X‐ray images. Type A: CBD < 3 cm; Type B: C7PL shifted to the concave side of the curve, and CBD > 3 cm; Type C: C7PL shifted to the convex side of the curve, and CBD > 3 cm.

### X‐ray Assessment

**FIGURE 1:**
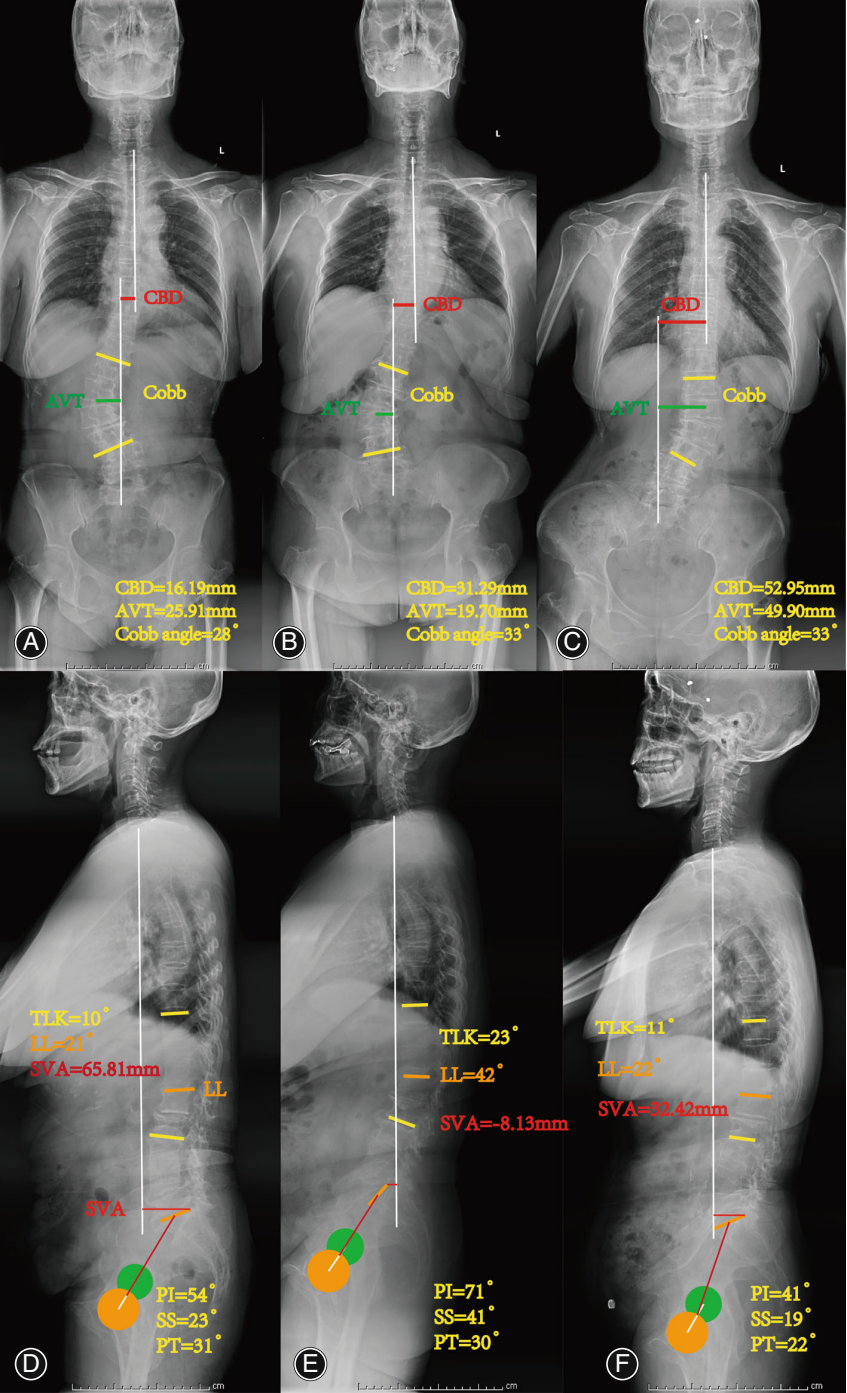
Radiographs showing three sub‐types: Type A (A, D) shows CBD 16.19 mm, AVT 25.91 mm, Cobb Angle 28°, TLK 10°, LL 21°, SVA 65.81 mm, SS 23°, PI 54°. Type B (B, E) shows CBD 31.29 mm, AVT 19.70 mm, Cobb Angle 33°, TLK 23°, LL 42°, SVA −8.13 mm, SS 41°, PI 71°; Type C (C, F) showed CBD 52.95 mm, AVT 49.90 mm, Cobb Angle 33°, TLK 11°, LL 22°, SVA 32.42 mm, SS 19°, PI 41°.

Bone structure parameters (Fig. 1), including Cobb angle, lumbar lordosis (LL), Loss of LL, CBD, thoracolumbar kyphosis (TLK), sacral slope (SS), pelvic incidence (PI), sagittal vertical axis (SVA), apical vertebral rotation (AVR), apical vertebral translation (AVT), direction of lateral bending, lumbar spondylolisthesis (LS) and T‐value were recorded and measured using neutral standing radiographs.^24^

### MRI Measurement

The lumbar spine was examined using a 1.5 T MR scanner (Signa HDxt 1.5T, GE Healthcare, Chicago, IL, USA). T2‐weighted (T2W) images were captured with TR/TE times of 2300 ms/99 ms, FA: 180, a matrix of 512 × 204, a 4 mm slice thickness, and a 1 mm intersection gap.

**FIGURE 2:**
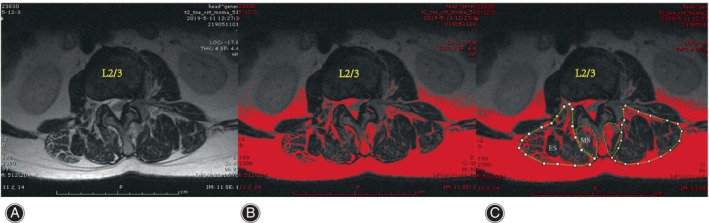
Muscle measurements using ImageJ. MS, multifidus; ES, erector spinae; FIA, fat infiltration area. Choosing the 8‐bit image (A). Using the threshold tool of the program, the red area was measured as FIA (B). The regions of interest were outlined with a graphic cursor (C).

**FIGURE 3:**
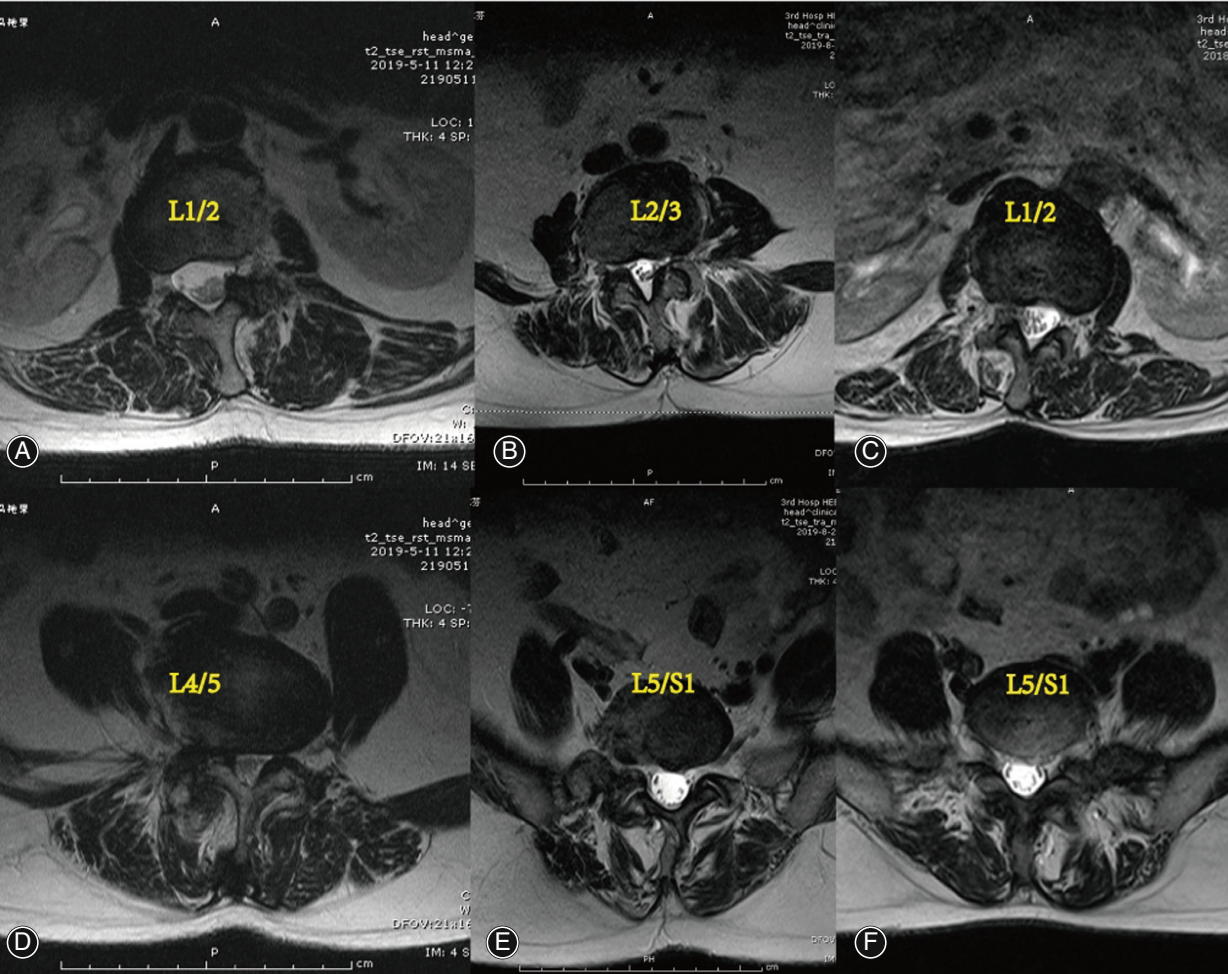
MRI showing the apex of the primary curve and fractional curve of the three sub‐types. Type A (A, D) shows L1/2, L4/5. Type B (B, E) shows L2/3, L5/S1. Type C (C, F) shows L1/2, L5/S1.

The cross‐sectional areas (CSAs) of the MS and ES muscles at the apex of the primary and fractional curves were determined by tracing the muscle's fascial boundary using Image J‐win64 software.^25^ To evaluate fat infiltration, the fat infiltration area (FIA) within the total CSA of both muscles was quantified using a threshold technique.^26^ Subsequently, the percentage of fat infiltration area (%FIA) relative to the total CSA was calculated (Fig. 2). The central image, corresponding to the apical vertebra, was selected for PM assessment (Fig. 3). The convex side encompassed both the primary curve's convex side and the compensatory curve's convex side, with the difference value between the concave and convex sides indicating the degree of asymmetric PM change. We defined the difference of more than 3% between concave and convex paravertebral muscle fat infiltration as asymmetrical threshold. Paravertebral muscle parameters and imaging parameters were measured respectively by two observers independently, blinded to the patients' characteristics.

### Statistical Analysis

The data were analyzed using SPSS 26.0 statistical software by IBM (Armonk, NY, USA). A Kolmogorov–Smirnov test was used to determine the normality of data distribution. Continuous variables with an approximately normal distribution are expressed as the mean and standard deviation (SD), and as the median (interquartile range) otherwise. A paired *t*‐test or one‐way analysis of variance (ANOVA) was used to analyze the difference of continuous variables, and the Mann–Whitney U test for non‐normally distributed data were used. An χ2 analysis and Fisher's exact test were applied to the categorical data. A *p*‐value of <0.05 was considered statistically significant. Linear regression analysis was utilized to analyze the correlation between bone structure parameters and the degree of asymmetry.

## Results

### Demographic and Radiographic Characteristics between Three Types

The primary curves consisted of thoracolumbar or lumbar curves. The additional curve included lumbosacral curves, and its direction was opposite to the primary curve. Type A was the most prevalent type, with more Type C patients than Type B patients (19.0% compared to 7.1%). There were no significant differences in terms of demographic and radiographic characteristics between three types as shown in Table 1. The number of vertebrae in the primary curve also showed no significant differences among the three subgroups (χ^2^ = 4.608, *p* = 0.294) as shown in Table 1. No significant differences were observed in terms of Cobb angle, PI, SS, PT, LL, TLK, SVA, AVT and LL loss, Kyphosis, LS, T‐value, L and R of main curve among the three subgroups in Table 2. However, the CBD in Type B and C patients was significantly greater than that in Type A.

**TABLE 1:** Demographic and radiographic characteristics between three types.

Subject		Type A	Type B	Type C	Statistics	*p*‐value
Number		62	6	16	‐	‐
Age (years)		62.42 (5.88)	63.67 (4.72)	63.00 (6.72)	F = 0.159	0.853
Gender, M:F (% of F)		6:56 (90.3)	1:5 (83.3)	1:15 (93.8)	χ^2^ = 0.522	0.770
BMI (kg/m^2^)		26.51 (3.62)	25.24 (4.60)	26.11 (3.83)	F = 0.357	0.701
Thoracolumbar curves (L:R)		12 (5/7)	1 (1/0)	2 (1:1)	χ^2^ = 0.441	0.802
Lumbar curves (L:R)		50 (17/33)	5 (3/2)	14 (5:9)		
Lumbosacral curves		62	6	16	‐	‐
AVL of main curve	L1‐L1/2	9	1	7	χ^2^ = 6.554	0.161
	L2‐L2/3	35	3	7		
	L3‐L3/4	18	2	2		
AVL of fractional curve	L4‐L4/5	10	2	3	χ^2^ = 1.272	0.866
	L5	20	1	5		
	L5/S1	32	3	8		
AVR of main curve (Nash–Moe)	I	34	4	8	χ^2^ = 0.974	0.957
	II	25	2	7		
	III	3	0	1		
Vertebrae number in main curve	3	16	0	7	χ^2^ = 4.608	0.294
	4	36	5	8		
	5	10	1	1		

Abbreviations: AVL, apical vertebral level; AVR, apical vertebral rotation.

**TABLE 2:** Radiographic summary of DLS patients between three types.

Subject	Type A	Type B	Type C	Statistics	*p*‐value
Cobb angle (°)	27.75 (8.14)	34.50 (4.32)	27.15 (5.91)	F = 2.329	0.104
PI (°)	49.67 (10.65)	49.50 (16.69)	52.32 (14.34)	F = 0.327	0.722
SS (°)	26.58 (11.32)	16.50 (14.78)	26.98 (13.98)	F = 1.977	0.145
PT (°)	23.09 (8.5)	28.17 (5.78)	24.09 (7.73)	F = 1.074	0.346
LL (°)	24.50 (26.05)	20.33 (13.13)	29.69 (15.39)	Z = 1.478	0.478
TLK (°)	10.00 (20.00)	27.83 (24.06)	10.53 (7.27)	Z = 3.274	0.195
SVA (mm)	53.27 (35.21)	68.23 (57.57)	55.05 (66.82)	Z = 0.337	0.845
AVT (mm)	28.20 (12.20)	26.43 (10.05)	34.74 (9.88)	F = 2.190	0.119
CBD (mm)	14.78 (13.36)	51.69 (19.24)	39.41 (18.91)	Z = 48.205	0.001*
LL loss	11	2	3	χ^2^ = 1.213	0.633
Kyphosis	13	1	2	χ^2^ = 0.539	0.886
LS	12	2	6	χ^2^ = 2.923	0.237
T‐value	−1.42 (1.16)	−1.40 (0.76)	−1.77 (0.99)	F = 0.646	0.527
L of main curve	22	4	6	χ^2^ = 2.222	0.336
R of main curve	40	2	10		

Abbreviations: L: R, left/right lateral bending; LS, lumbar spondylolisthesis.

* Significant if *p* < 0.05.

### %FIA and Asymmetric Degree of Paraspinal Muscles between Three Types

The mean %FIA was significantly higher on the concave side compared to the convex side at the apex of both the main and fractional curves in the multifidus and erector spinae muscles (*t* = −15.595, −8.818, *p* < 0.05; *t* = −11.361, −10.845, *p* < 0.05). Notably, it was significantly greater on the concave side of the multifidus in Type B and C compared to Type A, while no significant differences were observed between Type B and Type C in Tables 3 and 4. The degree of asymmetry was significantly greater in the multifidus at the apex of the main curve in Type B and Type C compared to Type A, with no significant differences between Type B and Type C in Table 5. Additionally, the degree of asymmetry was significantly greater in the multifidus at the apex of the fractional curve in Type B compared to Type A, with no significant differences between Type A and Type C (Table 5).

**TABLE 3:** %FIA of ES and MS on the concave and convex sides between three types at the apex of the main curve.

Subgroup	ES (%FIA)		MS (%FIA)	
	Convex side	Concave side	Convex side	Concave side
Type A	17.59 (7.31)	25.49 (10.38)	17.17 (6.62)	28.77 (4.87)
Type B	18.08 (4.95)	28.31 (4.28)	21.09 (4.60)	42.01 (9.69)
Type C	17.15 (6.65)	25.69 (11.90)	18.38 (7.63)	40.37 (15.47)
*F value*	0.043	0.206	1.307	9.046^a^
*P* value	0.958	0.814	0.276	0.005*

*Notes*: Analysis of variance test was performed.

* Significant if *p* < 0.05.

^a^ Welch test.

**TABLE 4:** %FIA of ES and MS on the concave and convex sides between three types at the apex of the fractional curve.

Subgroup	ES (%FIA)		MS (%FIA)	
	Convex side	Concave side	Convex side	Concave side
Type A	14.13 (6.88)	24.52 (9.04)	17.77 (6.88)	29.29 (8.60)
Type B	18.50 (4.90)	27.98 (4.89)	22.51 (6.36)	48.06 (10.42)
Type C	18.01 (7.96)	26.69 (9.80)	24.17 (10.43)	44.68 (15.35)
*F* value	2.689	0.683	3.636^a^	14.661^a^
*P* value	0.074	0.508	0.058	0.001*

*Notes*: Analysis of variance test was performed.

* Significant if *P* < 0.05.

^a^ Welch test.

**TABLE 5:** The asymmetric degree at the apex in ES and MS between three types.

Subgroup	Asymmetric degree at the main curve		Asymmetric degree at the fractional curve	
	ES (%FIA)	MS (%FIA)	ES (%FIA)	MS (%FIA)
Type A	7.29 (11.29)	12.57 (4.27)	8.23 (11.92)	11.51 (8.68)
Type B	10.10 (7.06)	20.73 (17.30)	8.46 (7.93)	25.56 (8.58)
Type C	6.39 (10.99)	20.37 (19.66)	6.18 (22.98)	20.51 (16.57)
Statistics	Z = 1.569	Z = 17.572	Z = 0.196	F = 8.459^a^
*P* value	0.456	0.001*	0.907	0.005*

* Significant if *p* < 0.05.

^a^ Welch test.

### The Correlation Analysis between the Asymmetric Degree of PM Change and Spinal Parameters

**FIGURE 4:**
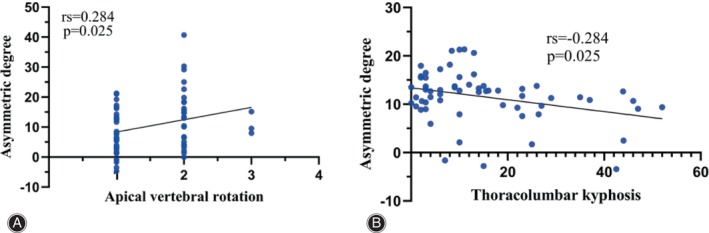
Graph showing the correlation between asymmetric degree in ES and AVR at the apex of the fractional curve (A), and that in MS and TLK at the apex of the main curve in Type A (B). ES, erector spinae; AVR, apical vertebral rotation; MS, multifidus; TLK, thoracolumbar kyphosis.

**FIGURE 5:**
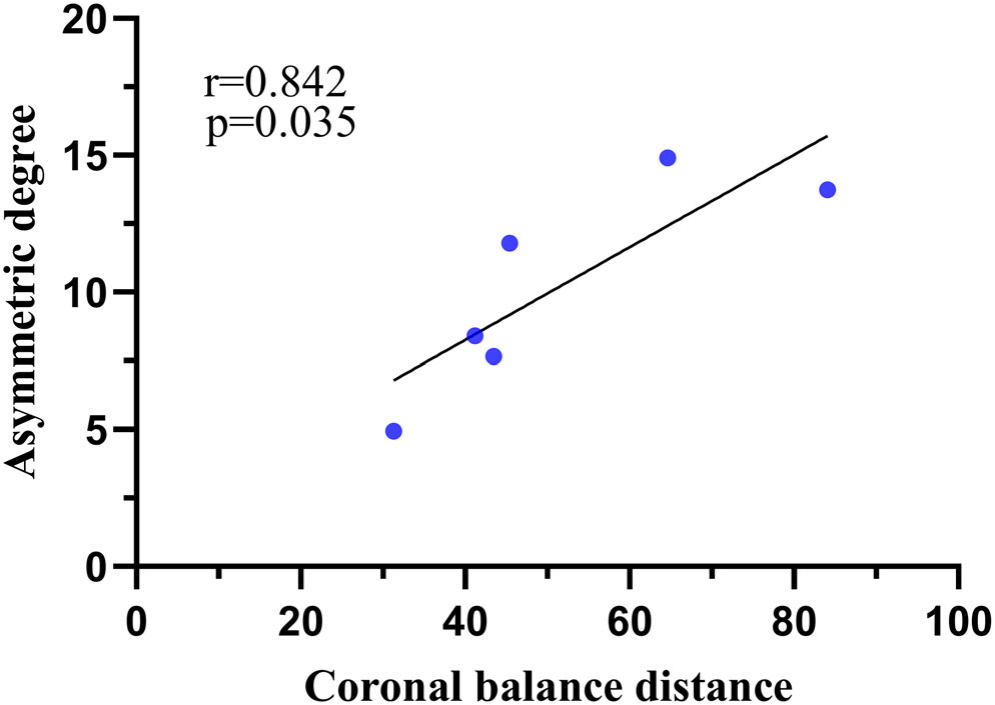
Graph showing the correlation between asymmetric degree in ES and CBD at the apex of the main curve in Type B. ES, erector spinae; CBD, coronal balance distance.

**FIGURE 6:**
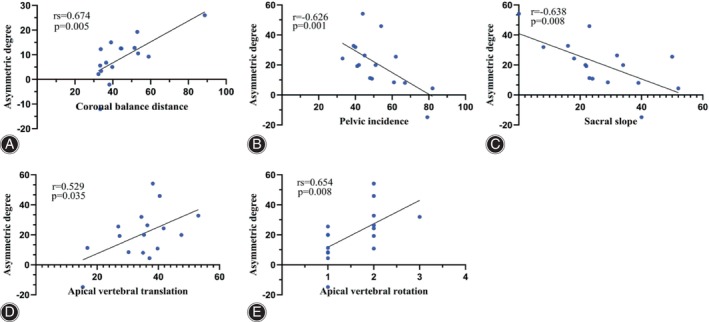
Graph showing the correlation between asymmetric degree in ES and CBD at the apex of the fractional curve (A), and that in MS and PI, SS, AVT, AVR at the apex of the fractional curve in Type C (B‐E). ES, erector spinae; CBD, coronal balance distance; MS, multifidus; PI, pelvic incidence; SS, sacral slope; AVT, apical vertebral translation; AVR, apical vertebral rotation.

The asymmetric degree (AD) in the change of the multifidus was significantly higher at the apex of both the main and fractional curves compared to the erector spinae (Z = ‐4.839, *p* < 0.05; Z = ‐2.695, *p* < 0.05). The change in AD of the erector spinae was weakly positively correlated with the AVR (0 < rs <1, *p* < 0.05), while that of MS was weakly negatively correlated with TLK in Type A (−1 < rs <0, *p* < 0.05) (Fig. 4). The change in AD of the erector spinae was strongly positively correlated with CBD at the apex of the main curve in Type B (0 < r < 1, *p* < 0.05) (Fig. 5). At the apex of the fractional curve, the change in AD of the erector spinae was strongly positively correlated with CBD (0 < rs <1, *p* < 0.05), and the change in AD of the multifidus was strongly positively correlated with AVR (0 < rs <1, *p* < 0.05), while it was strongly negatively correlated with PI and SS (−1 < r < 0, *p* < 0.05) in Type C (Fig. 6).

## Discussion

Our analysis of the results revealed patients in Type B and C demonstrated higher fat infiltration in MS on the concave side of both the main and fractional curves when compared to those in Type A. The asymmetric degree of ES change was positively correlated with CBD at the main curve in Type B and at the fractional curve in Type C, and that of MS was positively correlated with AVR, while negatively strong‐correlated with PI and SS in Type C.

### Morphology and Function of Multifidi and Erector Spinae

In recent times, there has been an increasing focus among researchers on the relationship between paraspinal muscles and lumbar degenerative diseases.^25^, ^27^ PMs play an important role in protecting spinal column from a sudden power.^28^ According to Gray *et al*.,^29^ the multifidus muscles are capable of lateral flexion, extension, and rotation. Macintosh and Bogduk^30^ found the multifidus muscles primarily contribute to the posterior sagittal rotation of the lumbar vertebrae. Regarding the erector spinae muscles, unilateral contractions cause lateral flexion of the vertebral column, while bilateral contractions produce posterior sagittal rotation. Consequently, these muscles are well‐suited to collaborate with the multifidus muscles in opposing the flexion effect of the abdominal muscles during trunk rotation.^31^ Therefore, we selected the MS and ES muscles as the subjects of our study.

### Characteristic of Paraspinal Muscle Change in Coronal Sub‐type of DLS

The mean %FIA were significantly higher on the concave side compared to the convex side at the apex of both curves in the multifidus and erector spinae muscles in this study, which aligns with previous reports.^13^, ^14^ Notably, patients in Type B and C exhibited higher levels of fat infiltration on the concave side of the multifidus muscle at the apex of both curves compared to those in Type A. There are three main reasons for this phenomenon: first, the multifidus muscle is particularly sensitive to pathological changes.^32^ Second, the segmental organization of the multifidus muscles anchors them to each lumbar vertebra below, including the mamillary processes, iliac crest, and sacrum.^31^, ^33^ Third, the greater CBD leads to a more asymmetric mechanical load between the convex and concave sides. These three factors are considered the primary contributors to the worsening of fatty infiltration in the multifidus muscle.

Although fatty deposits were also present in the ES, there was no significant difference in the ES among the three subtypes. This disparity between the MS and ES could be related to the following sections. There are differences in muscle innervation.^34^ The multifidus muscles are the primary muscles in the lumbosacral transition zone, crucial for posterior stabilization, whereas the erector spinae muscles mostly traverse the lumbar region without direct attachment to the lumbar vertebrae.^29^, ^33^ Regarding the connection between paraspinal muscle degeneration and lumbar stability, the degeneration of the multifidus muscles was more pronounced compared to the erector spinae muscles.^35^ These findings collectively support our perspective.

### Correlation Analysis and Interpretation of the Asymmetric Degree and Spinopelvic Parameters

It is noteworthy that in our study, the extent of asymmetry in ES change exhibited a strong positive correlation with the CBD in Type B and C. Furthermore, the asymmetry in fatty infiltration within the multifidus muscle exceeded that of the erector spinae in all three subgroups. Reduced density of paraspinal muscles is identified as a contributor to coronal imbalance.^23^ We posit that the relationship between the erector spinae and coronal spine stability becomes more pronounced following multifidus decompensation.

A prior study has established that the primary function of the multifidus muscle is to maintain the posterior sagittal rotational balance of the lumbar vertebrae.^30^ In our current investigation, we observed a strong positive correlation between the degree of asymmetry in multifidus muscle changes and the AVR. Notably, there exists a negative correlation between qualitative muscle changes caused by fat infiltration and muscle strength.^36^ The unopposed action of the deep spinal transverse and rotator muscles is a significant factor in initiating deformities in many idiopathic curves.^37^ We hypothesize that this mechanism also operates in DLS. Conversely, this asymmetry showed a strong negative correlation with the PI and SS in Type C. Paravertebral muscles attach to the sacrum as they pass between the lumbar vertebrae and ilium. It seems reasonable that the larger the size of the multifidus, the greater the strength, and thus the greater the PI and SS angle. As is well known, there exists a negative correlation between qualitative muscle changes caused by fat infiltration and muscle strength. Because of the negative correlation between fat infiltration and muscle strength, the correlation between the multifidus fat infiltration and PI or LL is logical. Minetama *et al*. and Menezes‐Reis *et al*.^11^, ^12^ identified that multifidus volume was positively correlated with PI and SS (I = 0.22; 0.08), and its fat infiltration was negatively correlated with PI (I = −0.11) in asymptomatic adults, in line with our findings. While they also found multifidus fat infiltration was positively correlated with SS (*r* = 0.15). The cause of the discrepancy with our results might be interpreted by the different diseases and study design. We believe that, in addition to coronal imbalance, these pelvic parameters can exacerbate paravertebral muscle degeneration.

It is not surprising that the degree of asymmetry in multifidus muscle changes positively correlated moderately with AVT in Type C, while the degree of asymmetry in erector spinae changes weakly correlated positively with AVR in Type A, as lateral vertebral translation often deviates from the concave side, and the unopposed action of deep spinal transverse and rotator muscles plays a significant role in these processes. Current guidelines stress the importance of addressing sagittal imbalance, but it is equally crucial not to disregard the coronal component. In our study, we observed that 26.2% of DLS patients displayed pre‐operative coronal imbalance among the 84 patients in our sample, which aligns with previous findings.^23^, ^38^ Based on the CBD, we categorized patients as having either coronal balance (CBD < 3 cm) or coronal imbalance (CBD > 3 cm).^23^, ^39^ In this investigation, Type B and Type C exhibited significantly greater CBD compared to Type A. Moreover, the asymmetrical degeneration of paraspinal muscles is linked to spine instability and may contribute to the progression of scoliosis.^6^ Conversely, spinal deformity may also play a role in the development of fat infiltration in paraspinal muscles.

### Strengths and Limitations

The strengths of this study are as follows. First, this study reports the degree of fatty infiltration in paraspinal muscles differs among various coronal subtypes of DLS patients for the first time. Second, based on the differences of PM among the three subgroups, our findings may help to make more rational treatment plans. The risk of more rigid fixation in surgery and postoperative development of malalignment might be considered. Of course, this study also has certain limitations. First, being a retrospective study, it cannot establish causality between morphological changes in paraspinal muscles and DLS. Second, the sample size, especially in Type B, was relatively small, potentially introducing bias into the results. Longitudinal studies with large‐sample sizes are necessary to elucidate the underlying causes of these morphological changes.

## Conclusions

In conclusion, the degree of fatty infiltration in paraspinal muscles differs among various coronal subtypes of DLS patients. The CBD in patients with coronal imbalance is correlated with the degree of asymmetry in erector spinae changes.

## Conflict of Interest Statement

The authors declare no competing interests.

## Ethics Statement

This retrospective study was performed in line with the principles of the Declaration of Helsinki. Approval was granted by the Ethics Committee of the Third Hospital of Hebei Medical University. (protocol code 2022‐094‐1; 2022/10/25).

## Author Contributions

Zhenguo Shang: data measurements and manuscript preparation; Hengrui Chang: data measurements and manuscript revision; Jiaxin Xu and Wenyuan Ding: manuscript revision; Di Zhang and Hui Wang: study design. All authors read and approved the final manuscript.
